# Vaccinia virus lacking the Bcl-2-like protein N1 induces a stronger natural killer cell response to infection

**DOI:** 10.1099/vir.0.2008/004119-0

**Published:** 2008-11

**Authors:** Nathalie Jacobs, Nathan W. Bartlett, Richard H. Clark, Geoffrey L. Smith

**Affiliations:** Department of Virology, Faculty of Medicine, Imperial College London, St Mary's Campus, Norfolk Place, London W2 1PG, UK

## Abstract

The vaccinia virus (VACV) N1 protein is an intracellular virulence factor that has a Bcl-2-like structure and inhibits both apoptosis and signalling from the interleukin 1 receptor, leading to nuclear factor kappa B activation. Here, we investigated the immune response to intranasal infection with a virus lacking the *N1L* gene (vΔN1L) compared with control viruses expressing *N1L*. Data presented show that deletion of *N1L* did not affect the proportion of CD4^+^ and CD8^+^ T cells infiltrating the lungs or the cytotoxic T-cell activity of these cells. However, vΔN1L induced an increased local natural killer cell activity between days 4 and 6 post-infection. In addition, in the absence of N1 the host inflammatory infiltrate was characterized by a reduced proportion of lymphocytes bearing the early activation marker CD69. Notably, there was a good correlation between the level of CD69 expression and weight loss. The implications of these findings are discussed.

*Vaccinia virus* (VACV) is a member of the genus *Orthopoxvirus* (OPV) of the *Poxviridae* (Moss, 2007; Smith, 2007). Like other OPVs, VACV has a double-stranded DNA genome of approximately 190 kb and encodes about 200 genes. Genes located in the left and right terminal regions of the genome are variable between OPVs and affect virulence, host range and immunomodulation. Gene *N1L* is located near the left end of the VACV strain Western Reserve (WR) genome and encodes a 14 kDa intracellular homodimer (Bartlett *et al.*, 2002). Bioinformatic analysis showed that the *N1L* gene is conserved in many OPVs (Bartlett *et al.*, 2002), despite being located in the terminal (variable) region of the genome. An exception to this conservation is the highly attenuated VACV strain modified virus Ankara (MVA) that encodes a truncated N1 protein (Antoine *et al.*, 1998). Overexpression of N1 in uninfected cells was reported to inhibit nuclear factor kappa B (NF-*κ*B) and interferon response factor 3 (IRF3) activation by binding to the inhibitor of kappa kinase (IKK) complex and TANK-binding kinase 1 (TBK1), respectively (DiPerna *et al.*, 2004). However, the crystal structure of N1 showed that this protein is a member of the Bcl-2 family of anti-apoptotic proteins (Aoyagi *et al.*, 2007; Cooray *et al.*, 2007) and it was demonstrated that N1 inhibited staurosporine-induced apoptosis in transfected and infected cells (Cooray *et al.*, 2007). N1 inhibits interleukin (IL)-1-induced NF-*κ*B activation in transfected cells (DiPerna *et al.*, 2004; Graham *et al.*, 2008) but this effect was not evident in infected cells (Cooray *et al.*, 2007), presumably due to the presence of other VACV NF-*κ*B signalling inhibitors. More recently, an additional study showed that N1 did not co-purify or co-precipitate with the IKK complex, unlike another VACV protein, B14 (Chen *et al.*, 2008).

VACV strains engineered to lack the *N1L* gene are attenuated in mice (Kotwal *et al.*, 1989; Bartlett *et al.*, 2002; Billings *et al.*, 2004). In an intradermal model of infection (Tscharke & Smith, 1999; Jacobs *et al.*, 2006) the deletion mutant, vΔN1L, induced smaller lesion sizes than wild-type (WT) and revertant (Rev) controls and less infectious virus was recovered from the infected tissue (Bartlett *et al.*, 2002). Similarly, vΔN1L was attenuated in the intracranial (Kotwal *et al.*, 1989) and intranasal model of infection (Bartlett *et al.*, 2002). However, the cellular immune response to infection with VACV lacking *N1L* has not been reported.

Here, we have investigated the effect of N1 on the host response after intranasal infection of female BALB/c mice (6–8 weeks old) with 10^4^ p.f.u. of WT VACV, a deletion mutant lacking *N1L* (vΔN1L) and a Rev virus (vN1-Rev) in which the *N1L* gene was reinserted into the *N1L* gene locus of vΔN1L (Bartlett *et al.*, 2002). After infection, groups of mice (*n*=6) were monitored daily for signs of illness and weights and, as noted previously (Bartlett *et al.*, 2002), animals infected with vΔN1L lost less weight than controls (data not shown). In addition, at different days post-infection (p.i.), the animals were sacrificed and the broncho alveolar lavage (BAL) fluids were prepared, and lungs, brain and spleen were removed. Cells present in the lungs were prepared as described previously (Clark *et al.*, 2006) and analysed after staining with appropriate combinations of fluorescein isothiocyanate (FITC)-, phycoerythrin (PE)-, allophycocyanine (APC)- or tricolour-labelled anti-CD3 (Caltag), anti-CD8 (BD Pharmingen), anti-CD4 (Caltag), anti-CD45 (pan leukocyte marker; BD Pharmingen) anti-CD25 (IL-2R*α*; BD Pharmingen), anti-CD69 (BD Pharmingen) or anti-pan NK (DX5; BD Pharmingen) antibodies. The presence of cell-surface markers was determined on a FACScan flow cytometer with CellQuest software (BD Biosciences) and a lymphocyte gate was used to select at least 20 000 events. There was no difference in the proportion of CD4^+^ or CD8^+^ T cells in the lungs at 3, 6 and 9 days p.i. with vΔN1L, compared to WT and Rev controls (data not shown). In addition, we measured the cytolytic activity of these cells by chromium release assays on VACV-infected P815 (VACV WR at 10 p.f.u. per cell for 2 h at 37 °C) as described in Clark *et al.* (2006) and found no difference between the groups at days 6 and 7 p.i. (data not shown). However, in three independent experiments there was an increased proportion of natural killer (NK) cells (CD3^−^ DX5^+^) in the lung (Fig. 1a[Fig f1]), and a similar increase was seen in BAL fluid (Fig. 1b[Fig f1]). These differences were seen consistently but with these sample sizes were not statistically significant. However, when the cytolytic activity of these cells was measured using Yac-1 target cells (Hussell & Openshaw, 1998), it was found that NK cells derived from infection with vΔN1L had significantly greater activity on day 4 p.i. compared with WT and Rev (*P*<0.05 comparing vΔN1L with WT and Rev, Student's *t*-test) (Fig. 1c[Fig f1]). This increased NK activity was observed only locally (lungs) and not in the spleen (data not shown).

Infection by vΔN1L induced an increased NK activity at early time points (day 4 p.i.) and therefore we measured if this affected the virus titres in the lungs at different times p.i. By 3 days p.i., the titres of all three viruses had increased to >10^6^ p.f.u. per lung and were indistinguishable from each other, showing that the N1 protein was not needed for efficient virus replication *in vivo*. However, consistent with the enhanced cytolytic activity of NK cells, by day 7 p.i. the titre of vΔN1L in the lung had started to fall, whereas the titres of control viruses were higher than on day 3. At this time point the difference between vΔN1L and both control viruses was significant (*P*<0.05). This difference between vΔN1L and controls was increased further by day 10 and at this time the titres of all viruses were starting to fall (Fig. 2). Data shown are the mean values from two independent experiments that gave the same result. This observation shows that although vΔN1L can replicate to high titres in mouse tissue, it is cleared more rapidly by the host immune response, consistent with its attenuated phenotype. The increased NK cell activity following infection with vΔN1L may partly explain the reduced virus titres seen at 7 days p.i. However, given the multiple roles of N1 in inhibiting both apoptosis and activation of NF-*κ*B via the IL-1R–TRAF6 pathway, it is probable that other factors may also be involved.

Next, we measured the activation status of the lymphocytes that infiltrated the lung. To do this the percentage of lymphocytes expressing the early activation marker CD69 was measured by FACS (anti-CD69-PE; BD Biosciences). At days 4 and 6 p.i. with WT and Rev virus, ∼20 % of lung lymphocytes expressed CD69 and this percentage decreased as the mice recovered from infection (day 11 p.i.) (Fig. 3a[Fig f3]). However, on days 4 and 6 p.i. with vΔN1L there was a significantly smaller proportion of lymphocytes (both NK and T cells) that was CD69^+^ compared with the proportion following infection with WT and Rev viruses (Fig. 3a[Fig f3]). This suggests that N1 somehow contributes to immune activation, and, conversely, in the absence of N1 there was a reduced immune activation. In the intranasal model of infection used here, the degree of weight loss correlates with the severity of pneumonia, which is promoted by excessive inflammation. Consequently, diminishing the inflammatory response can reduce illness. This was exemplified by the observation that expression of a secreted CC chemokine-binding protein from VACV strain WR decreased virus virulence in this model. This virus induced less weight loss, decreased virus titres and a reduced recruitment of inflammatory cells into the lungs (Reading *et al.*, 2003).

When the degree of weight loss was compared with CD69 expression it was found that there was a direct relationship: animals that lost more weight (infected by WT and Rev viruses) had a greater percentage of CD69^+^ cells, while those infected by vΔN1L showed lower weight loss and had a smaller percentage of CD69^+^ cells (Fig. 3b[Fig f3], *P*<0.0001). Although previous *in vitro* data suggest that CD69 exerts a pro-inflammatory function, recent *in vivo* results indicate that CD69 might act as a regulatory molecule, modulating the inflammatory response (Sancho *et al.*, 2005). A link between NK activity and CD69 expression was suggested by the observation that, compared with WT mice, CD69^−/−^ mice showed an enhanced NK-mediated anti-tumour response that led to greater protection and rejection of major histocompatibility complex class I low tumour cells (Esplugues *et al.*, 2003). Moreover, antibody against murine CD69, which downregulated CD69 expression, induced NK cytotoxic activity (Esplugues *et al.*, 2005). A relationship between CD69 and Bcl-2 has also been reported. In asthma, eosinophils in BAL expressed CD69 and antibody against human CD69 induced Bcl-2-dependent apoptosis (Foerster *et al.*, 2002).

An interesting parallel to the results reported here with the VACV N1 protein is the reduction in CD69 activation on B cells, which correlated with a decrease in splenomegaly, following infection with murine herpes virus 68 engineered to lack a viral Bcl-2 protein (vBcl-2) (de Lima *et al.*, 2005). Like N1, vBcl-2 is a Bcl-2 family protein that inhibits apoptosis (Wang *et al.*, 1999; Bellows *et al.*, 2000; Ku *et al.*, 2008). Moreover, it was suggested that B cells expressing higher levels of CD69 played a role in viral latency and this role was not directly linked to viral infection of these cells since only a minority of CD69^+^ B cells were infected (Krug *et al.*, 2007). Another viral anti-apoptotic Bcl-2 protein, M11L from myxoma virus (Kvansakul *et al.*, 2007) also plays a role in inflammation (Opgenorth *et al.*, 1992). While there are parallels between N1, M11 and vBcl-2 in that all these viral proteins are anti-apoptotic, a notable difference is that N1 also inhibits activation of NF-*κ*B, resulting from IL-1R signalling. In accord with this, infection of macrophages *in vitro* with VACV lacking gene *N1L* induced the production of pro-inflammatory cytokines, such as alpha and beta interferon, but also immunosuppressive cytokine such as IL-10 (Zhang *et al.*, 2005), suggesting that N1 regulates the inflammatory response in favour of the virus. Moreover, it was described in a tumour model that inhibition of NF-*κ*B in tumour cells induced an upregulation of CD69 on NK co-cultivated with these cells (Jewett *et al.*, 2006).

In conclusion, we demonstrate that deletion of the *N1L* gene from VACV strain WR causes reduced weight loss and virus titres in the lungs of mice infected intranasally. In this model, loss of N1 promotes a stronger NK cell response to infection but there are fewer CD69^+^ cells. Therefore, N1 can modulate both the NK response and lymphocyte activation. It remains to be determined whether these effects are due to the ability of N1 to inhibit apoptosis or signalling pathways, leading to NF-*κ*B activation.

## Figures and Tables

**Fig. 1. f1:**
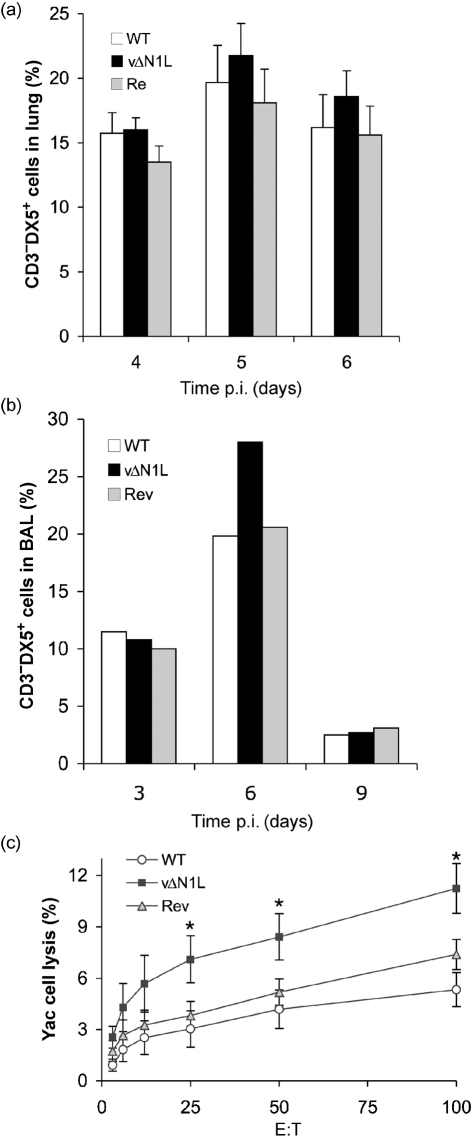
NK cells after intranasal infection with WT, vΔN1L and Rev. (a) The percentages of NK cells (% of CD3^−^DX5^+^ in lymphocyte gate) in the lung. Data shown are means±sem of four experiments. (b) Percentages of NK cells (% of CD3^−^DX5^+^ in lymphocyte gate) in BAL of one experiment with pooled data from four mice. (c) Chromium release assay of cells isolated from infected lung against NK-sensitive Yac cells. Data shown are means±sem of two experiments (E : T=effector : target ratio; the test is measured in triplicate).

**Fig. 2. f2:**
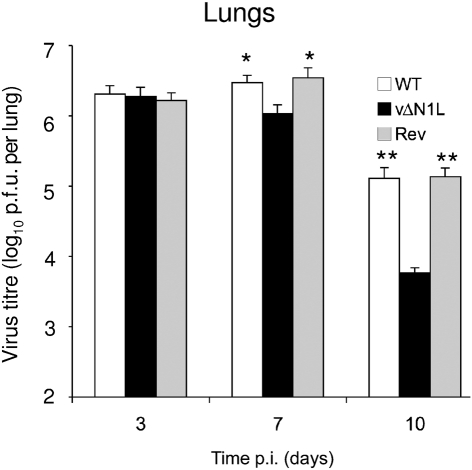
The N1L protein contributes to virulence in an intranasal infection model. Groups of five female BALB/c mice (6–8 weeks of age) were infected intranasally with 10^4^ p.f.u. of the indicated virus. On days 3, 7 and 10 lungs were harvested and assayed for infectious virus by plaque assay. The data are expressed as means±sem. The data were analysed by log-transforming followed by one-way ANOVA. Bonferroni's post-test with 95 % confidence levels (Prism 4 GraphPad) was used to pin-point significant differences. *, *P*<0.05; **, *P*<0.01 for vΔN1L compared with WT and Rev.

**Fig. 3. f3:**
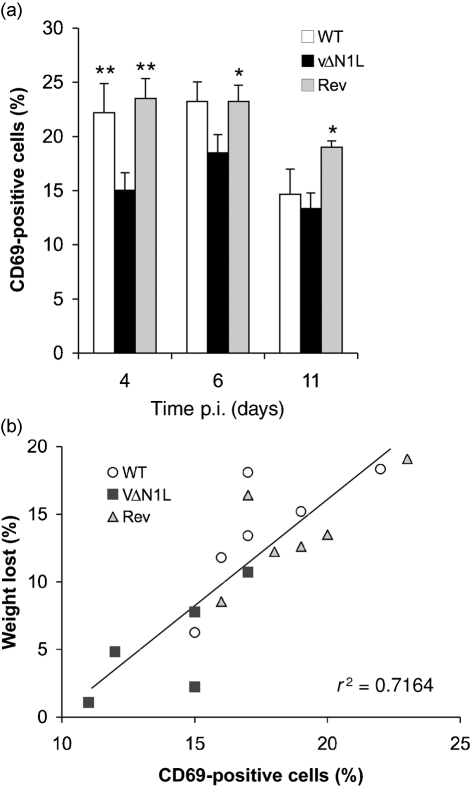
CD69 expression on lung cells after intranasal infection with WT, vΔN1L and Rev. (a) CD69 expression in lung (lymphocytes gate). Data are means±sem of two independent experiments. *, *P*<0.05; **, *P*<0.01. (b) Correlation between per cent weight loss and per cent of CD69-positive cells in the lungs at day 6 p.i. *P*<0.0001.
